# Analysis of the risk spillover network of G20 stock markets based on transfer entropy and complex network approaches

**DOI:** 10.1371/journal.pone.0336904

**Published:** 2025-12-01

**Authors:** Yijiang Zou, Qinghua Chen, Jihui Han, Longfeng Zhao

**Affiliations:** 1 School of Economics, Anyang Normal University, Anyang, Henan, China; 2 School of Systems Science, Beijing Normal University, Beijing, China; 3 School of Computer Science and Technology, Zhengzhou University of Light Industry, Zhengzhou, Henan, China; 4 School of Management, Northwestern Polytechnical University, Xi’an, Shaanxi, China; PLoS ONE, UNITED STATES OF AMERICA

## Abstract

This study investigates the risk spillover network among major stock market indices of G20 countries from 2003 to 2024. Transfer entropy is employed to measure the asymmetric and nonlinear information flow between stock markets. Based on this key metric, a directed and weighted risk spillover network among stock markets is constructed using the threshold method during periods of extreme events. Utilizing complex network theories, such as PageRank and betweenness centrality, the study analyzes the macro-topological characteristics of the risk spillover network and identifies key nodes. The findings not only demonstrate strong information interaction among G20 stock markets but also show that European and North American markets exhibit regional clustering characteristics, while emerging markets serve as bridging nodes in the risk spillover network. These results offer theoretical and practical insights for portfolio management, risk monitoring, and cross-border financial regulation and crisis management.

## 1 Introduction

In the context of global economic integration and highly interconnected financial markets, risk spillover effects have become a crucial topic in financial research. Volatility spillover effects frequently occur in an integrated economic system. Some researchers have found that recent financial instability worldwide mainly originates from certain developed and emerging markets [1]. The G20 countries encompass both developed and emerging economies, and their financial markets play a dominant role in the global economy.

According to data from the International Monetary Fund (IMF), the G20 economies account for more than 85% of global GDP, and their stock markets represent over 70% of total global market capitalization [2]. Therefore, the volatility of G20 stock markets not only affects their domestic economies but can also propagate to global financial markets [3]. Particularly, in the context of heightened global economic uncertainty, studying these risk spillovers helps reveal transmission pathways of global financial risks, identify key sources of risk, and provide policy recommendations for maintaining global financial stability.

Existing research primarily relies on linear models or traditional causality methods to analyze risk spillovers; however, these approaches are limited in capturing nonlinear propagation, directional information flow, and extreme market events. To address these gaps, this study combines the transfer entropy method with complex network theory to construct a directed weighted risk spillover network for G20 stock markets. Transfer entropy enables the quantification of nonlinear and directional information flows, accurately capturing risk transmission paths, while complex network analysis reveals key nodes and the overall network structure, identifying areas of concentrated risk and potential systemic vulnerability.

This study contributes to the literature in several ways. By integrating transfer entropy with complex network theory, it provides a nonlinear, directional, and structured analysis of G20 stock market risk spillovers, overcoming the limitations of traditional linear models. It systematically examines both the overall sample period and periods of extreme events, identifying key risk nodes and revealing the network structure of risk propagation, thus offering empirical evidence for portfolio management, risk monitoring, and financial stability assessment. Moreover, the study uncovers the roles of different markets in risk transmission and highlights areas of systemic risk concentration, providing practical guidance for cross-border financial regulation and crisis management.

This paper is organized as follows: Sect 2 provides a literature review on stock market risk spillovers, highlighting the use of transfer entropy and complex network approaches. Sect 3 introduces the methodologies and theories employed in this study. Sect 4 describes the data sample and presents descriptive statistical analysis. Sect 5 analyzes the risk spillover networks during the full sample period and periods of extreme events, respectively. Finally, Sect 6 discusses the mechanisms of risk propagation, theoretical implications, and policy relevance, while Sect 7 concludes by summarizing the main findings, contributions, and implications for portfolio management, financial stability, and cross-border regulation.

## 2 Literature review

The volatility of risk spillovers between stock prices or returns is a common phenomenon in stock markets. Related research holds significant theoretical and practical implications in areas such as efficient market theory, risk control, and portfolio construction. Generally, volatility relationships between stocks can be classified into synchronous relationships and causal relationships, including price co-movement [4], dependence [5], and risk contagion [6], among others. The verification and measurement of these relationships’ existence and magnitude are essential topics in stock market research, utilizing metrics such as *R*^2^ [7], Granger causality [8], and other various indicators and their derivatives [9,10].

The measurement of volatility is mostly achieved through the statistical relationships between stock return data, and transfer entropy has been proven to effectively quantify such statistical characteristics [11–13]. The information flow measurement based on transfer entropy is directional, capable of capturing the nonlinear characteristics of time series, and overcomes the weak quantification capability of Granger causality, making it widely applied in stock markets.

For instance, Oh *et al*. (2014) [14] and Bekiros [15] employed transfer entropy to measure the varying intensity of information flow before and after stock market crises. Sandoval *et al*. [16] and Kwon and Oh [17] suggested that information flow between stock indices of different countries, as well as between a national stock index and individual stocks, exhibits asymmetry. Dimpfl and Peter [18] utilized transfer entropy to quantify information flow between financial markets and proposed a bootstrap procedure suitable for statistical inference, allowing for the determination, measurement, and testing of information transfer without being constrained by linear dynamics.

Sensoy *et al*. [19] employed an effective transfer entropy symbolic encoding method to investigate the strength and direction of information flow between exchange rates and stock prices in several emerging economies. Niu [20] combined transfer entropy from information theory with a multi-scale analysis technique—complete ensemble empirical mode decomposition with adaptive noise—to innovatively measure the information transmission and correlation between China’s stock market and commodity futures market across different time scales. Shternshis *et al*. [21] developed a multi-step filtering approach to eliminate seasonality in volatility and heteroskedasticity, detect and remove spurious effects caused by stale prices, and filter out microstructure noise. These studies demonstrate that transfer entropy is highly effective in measuring the spatiotemporal characteristics of stock market information flow in terms of intensity and direction before and after critical events. Jia [22], in the study of global stock market risk spillovers, for the first time introduced the effective Rényi transfer entropy combined with wavelet analysis to systematically examine the tail risk spillover characteristics of global equity markets, revealing that the U.S. and European markets act as primary information sources, whereas Asian markets primarily serve as information recipients. Zou *et al*. [23] employed a time-varying parameter vector autoregression (TVP-VAR) model in conjunction with transfer entropy to investigate volatility spillover effects between Chinese and RCEP member countries’ stock markets, highlighting the responses to external risks in a complex international environment. Li *et al*. [24] utilized a transfer entropy-based framework to analyze risk diffusion and network resilience among ten emerging market countries, finding that Brazil, Mexico, and Saudi Arabia serve as the main risk transmitters, while India, South Africa, and Indonesia primarily function as risk recipients.

Since the introduction of the small-world network model [25] and the scale-free network model [26], complex networks have become a powerful tool for studying complex systems involving a large number of entities, including stock markets. In recent years, complex networks have been widely applied in various fields such as energy economics [27–31], international trade [32–34], financial markets [35–38], and cross-shareholding networks [39–41].

The spillover relationships among G20 countries can be represented as a complex network, where nodes correspond to countries and edges represent the risk spillover effects between their stock markets. Complex network theory provides useful tools for identifying key nodes, detecting clusters, and analyzing the overall topology of such spillover networks. Lai and Hu [42] estimated the Granger causality relationships among the stock markets of 20 countries from August 2019 to March 2020 and constructed a global stock market complex network based on the data. By comparing various characteristics of the topological structure of complex financial networks, they identified financial risks through centrality measures during stable and volatile periods. Wang *et al*. [43] constructed static and dynamic multilayer information spillover networks using daily returns from 24 publicly listed financial institutions in China from 2008 to 2018, analyzing the similarity, uniqueness, and overlap of different layers. Wen *et al*. [44] applied complex network analysis to investigate the extreme risk spillover effects between traditional financial institutions and fintech firms in the United States . Gong *et al*. [45] studied the tail risk contagion effects, dynamic characteristics, and cyclical patterns between the international energy and geopolitical multilayer networks based on complex network theory. Korkusuz *et al*. [46] combined the GARCH-BEKK model with complex network theory to analyze volatility spillover effects between global financial indicators and G20 stock markets. The study found that during crisis periods, network density significantly increases, resulting in faster and more direct risk transmission. An *et al*. [47] developed an early warning framework that integrates complex network models and machine learning to predict regime switches in complex financial systems. By analyzing the spillover network structure in multivariate time series, the authors were able to identify potential signals of regime shifts, providing preemptive insights for financial market stability. Gong *et al*. [48] investigated the cross-contagion mechanisms between international stock markets and geopolitical risks from a complex network perspective. The study revealed that geopolitical conflicts intensify risk contagion among international stock markets and that there are significant differences in risk transmission between developed and emerging markets.

This study employs the transfer entropy method to measure the risk spillover effects among G20 stock markets. Based on the analysis of static risk spillover effects, a directed weighted risk spillover network is constructed. Furthermore, key risk nodes in the risk spillover network during periods of extreme events are identified, extending the application of transfer entropy and complex network theory in portfolio construction, risk monitoring, and control in stock markets.

## 3 Methodology

### 3.1 Transfer entropy

In information theory, the uncertainty or information content of a discrete random variable can be measured using entropy, denoted as *H*(*X*). The magnitude of this measure is uniquely determined by the probability distribution *p*(*x*) of *X*, and is defined as follows:

$$H(X)=-\sum_{x}p\left(x\right)\log_{2}p\left(x\right).$$ (1)

where *X* denotes a discrete random variable, and *p*(*x*) is the probability that *X* takes the value *x*.

For a pair of discrete random variables (*X*,*Y*) with a joint probability distribution *p*(*x*,*y*), the joint entropy is given by

$$H(X,Y)=-\sum \sum p(x,y)\log_{2}p(x,y).$$ (2)

Given *X*, the uncertainty of *Y*, known as the conditional entropy of *Y*, is defined as:

$$H(Y|X)=-\sum_{x}\sum_{y}p\left(x,y\right)\log_{2}p\left(y|x\right).$$ (3)

Based on the fundamental concepts of entropy and information theory developed by Shannon [49], Schreiber [50] proposed the concept of “Transfer Entropy” (TE) to quantify asymmetric information transfer between subsystems.

First, the information flow from one process to another is defined by Eq (1), representing the reduction in the uncertainty of *X* given knowledge of *Y*, illustrating the role of *Y* in predicting *X*. Second, by comparing the historical information of *X* alone with the combined historical information of both *X* and *Y*, the contribution of *Y* to predicting *X* is quantified using the following formula:

$${TE}_{Y\to X}(k,l)=H_{X}(k)-H_{XY}(k,l)\;=\sum_{x_{n+1},x_{n}^{\left(k\right)},y_{n}^{\left(l\right)}}p\left(x_{n+1},x_{n}^{\left(k\right)},y_{n}^{\left(l\right)}\right)\log_{2}\frac{p\left(x_{n+1}|x_{n}^{\left(k\right)},y_{n}^{\left(l\right)}\right)}{p\left(x_{n+1}|x_{n}^{\left(k\right)}\right)}.$$ (4)

Here,

$$H_{X}(k)=\sum_{x_{n+1},x_{n}^{\left(k\right)},y_{n}^{\left(l\right)}}p\left(x_{n+1},x_{n}^{\left(k\right)},y_{n}^{\left(l\right)}\right)\log_{2}p\left(x_{n+1}|x_{n}^{\left(k\right)}\right),$$ (5)

which denotes the conditional entropy of *X* given its *k*-th order time-lagged subsequence $x_{n}^{\left(k\right)}=(x_{n},\cdots ,x_{n-k+1})$.

$$H_{XY}(k,l)=\sum_{x_{n+1},x_{n}^{\left(k\right)},y_{n}^{\left(l\right)}}p\left(x_{n+1},x_{n}^{\left(k\right)},y_{n}^{\left(l\right)}\right)\log_{2}p\left(x_{n+1}|x_{n}^{\left(k\right)},y_{n}^{\left(l\right)}\right)$$ (6)

represents the conditional entropy given both the *k*-th order time-lagged subsequence of process *X* and the *l*-th order time-lagged subsequence of process *Y*, where $y_{n}^{\left(l\right)}=(y_{n},\cdots ,y_{n-l+1})$.

Therefore, the calculation of transfer entropy requires the determination of the probability distribution of the random variables based on the values of *k* and *l*. Typically, the return series is divided into complete event groups, i.e., independent and complete states. The number of states or the box size varies in existing literature depending on the research objective. If global information is the focus, the sample frequency in each state should be the same, or equal-sized boxes should be used for interval division. If the focus is on extreme tail events, larger and smaller quantiles should be used, such as the 5% and 95% quantiles. In this paper, we use the 20%, 40%, 60%, and 80% quantiles (denoted as $q_{02},q_{04},q_{06},q_{08}$) to divide the return intervals into five states, with the state set being $s=\{s_{1},s_{2},s_{3},s_{4},s_{5}\}$ [11,18]. For reproducibility, all computations of transfer entropy in this study were performed in R (version 4.3.2) using the RTransferEntropy package, which provides nonparametric estimation of transfer entropy based on symbolic encoding.

### 3.2 Risk spillover measurement

To quantify the risk spillovers among the stock markets of the 19 G20 member countries, this study employs the transfer entropy (TE) method [50]. Transfer entropy captures the directed and potentially nonlinear flow of information between time series, making it suitable for modeling the propagation of financial risk across markets. Using the TE values, we define four categories of risk information flow to describe each country’s role in the network [51,52].

Risk information flow out measures the intensity of risk information transmitted from one country to another. For country *i* to country *j*, it is calculated as:

$${\text{Flow Out}}_{i\to j}=TE_{ij},$$ (7)

where *TE*_*ij*_ denotes the transfer entropy from country *i* to country *j*, capturing the amount of risk information transmitted from *i* to *j* [12].

Risk information flow in measures the intensity of risk information received by a country from others. For country *j* receiving risk information from country *i*, it is calculated as:

$${\text{Flow In}}_{j\to i}=TE_{ji},$$ (8)

where *TE*_*ji*_ represents the transfer entropy from country *j* to country *i*, indicating the amount of risk information received by *i* from *j* [53].

Net risk information flow out represents the difference between the risk information transmitted by a country and the risk information it receives. For country *i*, it is calculated as:

$${\text{Net Flow Out}}_{i}=\sum_{j\neq i}TE_{ij}-\sum_{j\neq i}TE_{ji},$$ (9)

where the first term $\sum_{j\neq i}TE_{ij}$ is the total risk information transmitted from country *i* to all other countries, and the second term $\sum_{j\neq i}TE_{ji}$ is the total risk information received by country *i*. A positive value indicates that country *i* is a net risk exporter [51].

Net risk information flow in is the converse measure, representing the extent to which a country is a net risk importer. For country *i*, it is calculated as:

$${\text{Net Flow In}}_{i}=\sum_{j\neq i}TE_{ji}-\sum_{j\neq i}TE_{ij},$$ (10)

where $\sum_{j\neq i}TE_{ji}$ is the total risk information received by country *i*, and $\sum_{j\neq i}TE_{ij}$ is the total risk information transmitted by country *i*. A positive value indicates that country *i* is a net risk importer [51,52].

### 3.3 Risk spillover network

In the stock market risk network, the node set *V* of the network G=(V,E) represents the stock indices of different countries, while the edge set *E* represents some form of association between the stock markets of different countries. In this paper, this association is represented by the risk spillover between the countries, with the weights denoted by the set *W*. From the definition, the initial network constructed from the transfer entropy matrix is a fully connected, directed, and weighted network, meaning that each country’s stock market exhibits bidirectional risk spillovers with every other country (excluding itself). To filter out redundant information, after setting the threshold *θ*, the rules for determining the risk spillover edge *e*_*ij*_ from country *i* to country *j* and its corresponding weight *w*_*ij*_ are given by Eqs (11) and (12). When *e*_*ij*_ = 1, it indicates the existence of an edge from country *i* to country *j*, and the weight is *w*_*ij*_ = *TE*.

$$E=\left\{ \begin{array}{cc} e_{ij}=1 & \;\text{if }i\neq j\text{ and }TE_{ij}\geq \theta \\ e_{ij}=0 & \;\text{otherwise} \end{array}\right.$$ (11)

$$W=\left\{ \begin{array}{cc} w_{ij}=TE & \;\text{if }i\neq j\text{ and }TE_{ij}\geq \theta \\ w_{ij}=0 & \;\text{otherwise} \end{array}\right.$$ (12)

The advantages of using complex network theory to study the stock market are reflected in two aspects: at the micro level, it can identify key stock nodes within the entire stock market; at the macro level, it can capture the topological structure of the correlations between different countries’ stock markets. Below is a brief introduction to the weighted network topology indicators used in this paper when measuring the risk spillover relationships in the stock market.

In a weighted directed network, the in-degree is the sum of the weights of all incoming edges of a node. Let *w*_*ji*_ represent the weight of the edge from node *j* to node *i* (here,the transfer entropy value), then the weighted in-degree of node *i* is:

$$k_{i}^{\text{in}}=\sum_{j=1}^{n}w_{ji}.$$ (13)

where *n* is the number of nodes directly connected to node *i*.

To compare the importance of nodes in networks of different sizes, the weighted in-degree is normalized as:

$$C_{\text{in}}(i)=\frac{k_{i}^{\text{in}}}{\sum_{i=1}^{N}\sum_{j=1}^{N}w_{ji}}.$$ (14)

where *N* is the total number of nodes in the network, and $C_{\text{in}}(i)\in [0,1]$. A larger $C_{\text{in}}(i)$ indicates that node *i* receives more spillovers from other markets.

Similarly, the weighted out-degree of node *i* is the sum of the weights of all outgoing edges:

$$k_{i}^{\text{out}}=\sum_{j=1}^{n}w_{ij}.$$ (15)

The normalized weighted out-degree centrality is:

$$C_{\text{out}}(i)=\frac{k_{i}^{\text{out}}}{\sum_{i=1}^{N}\sum_{j=1}^{N}w_{ij}}.$$ (16)

where a larger $C_{\text{out}}(i)$ indicates that node *i* has a stronger risk spillover effect on other nodes.

PageRank is a web ranking algorithm used to measure the importance of nodes in a directed network, which can be naturally extended to weighted directed networks by using the weighted adjacency matrix *W* = [*w*_*ij*_]. The weighted PageRank of node *i* is defined as:

$$PR(i)=\frac{1-d}{N}+d\sum_{j\in M(i)}\frac{w_{ji}}{\sum_{k}w_{jk}}\;PR(j),$$ (17)

where *N* is the total number of nodes in the network, *d* is the damping factor, *M*(*i*) is the set of nodes pointing to *i*.

In matrix form:

$$PR=(1-d)\frac{𝐞}{N}+d{𝐖}^{T}{𝐃}_{w}^{-1}PR,$$ (18)

where $𝐖=[w_{ij}]$ is the weighted adjacency matrix, and ${𝐃}_{w}=\text{diag}\;(\sum_{k}w_{1k},\ldots ,\sum_{k}w_{Nk})$ is the diagonal matrix of weighted out-degrees.

Betweenness centrality in a weighted network is calculated based on weighted shortest paths. To transform edge weights into distances, we set $d_{ij}=1/w_{ij}$ (if *w*_*ij*_ = 0, then $d_{ij}=+\infty$). The weighted betweenness centrality of node *i* is defined as:

$$C_{B}(i)=\sum_{\begin{array}{c} j\neq i\neq k\\ j,k\in V \end{array}}\frac{\sigma_{jk}^{w}(i)}{\sigma_{jk}^{w}},$$ (19)

where $\sigma_{jk}^{w}$ is the number of weighted shortest paths from node *i* to node *k*, and $\sigma_{jk}^{w}(i)$ is the number of such paths passing through node *i*. A higher *C*_*B*_(*i*) indicates a stronger ability of node *i* to act as an intermediary in risk transmission.

## 4 Data and descriptive statistics

This study selects the stock indices of the major stock markets of the G20 countries as sample data, with a time span from January 2, 2003, to December 31, 2024, covering representative stock indices from 19 countries. All selected data are daily closing prices. Since the European Union, as a G20 member, does not have a unified stock index, and its main member countries such as France and Germany have been included separately, the sample does not include data for the EU as a whole. The selected indices include Argentina’s MERV, Australia’s AUS200, Brazil’s BOVESPA, Canada’s GSPTSE, China’s SSE, France’s CAC40, Germany’s DAX30, India’s SENSEX, Indonesia’s JKSE, Italy’s FTMIB, Japan’s Nikkei225, South Korea’s KOSPI, Mexico’s IPC, Russia’s MICEX, Saudi Arabia’s TASI, South Africa’s TOP40, Turkey’s ISE100, the UK’s FTSE100, and the US’s SP500, as shown in Table 1. These indices are core indicators of each country’s stock market and can comprehensively reflect the stock market performance of major global economies. All data are sourced from the Wind database.

**Table 1:** The 19 sample countries and corresponding stock indexes of G20 countries.

No.	Country	Stock Index	No.	Country	Stock Index
1	Argentina	MERV	11	Japan	Nikkei225
2	Australia	AUS200	12	South Korea	KOSPI
3	Brazil	BOVESPA	13	Mexico	IPC
4	Canada	GSPTSE	14	Russia	MICEX
5	China	SSE	15	Saudi Arabia	TASI
6	France	CAC40	16	South Africa	TOP40
7	Germany	DAX30	17	Turkey	ISE100
8	India	SENSEX	18	UK	FTSE100
9	Indonesia	JKSE	19	USA	SP500
10	Italy	FTMIB			

Following standard practice in financial econometrics, we first remove missing values and align trading days across countries, resulting in a balanced panel with 5,342 observations for each market. Then, stock returns are calculated as $R_{t}=\ln x_{t}\;-\;\ln x_{t-1}$, where *R*_*t*_ represents the return on the *t*-th trading day, *x*_*t*_ is the stock closing price on the *t*-th trading day, and *x*_*t*−1_ is the stock closing price on the (*t*–1)-th trading day.

Table 2 presents the descriptive statistics of the return data. From the mean, the daily average return of the stock markets of various countries is generally low, with Argentina having the highest daily average return and Italy having the lowest, indicating significant differences in the return performance of stock markets across countries. Regarding the distribution characteristics of the returns, most countries’ return series show negative skewness, especially Argentina and Russia, suggesting that extreme negative return events are more frequent in these markets. In addition, the return series of all countries exhibit peak characteristics, with Russia having the highest kurtosis value, indicating that its return distribution has a fatter tail and a higher probability of extreme events. The Jarque-Bera test results further confirm that the return distributions of all countries significantly deviate from normal distribution, which may be due to the frequent occurrence of volatility clustering and extreme events in financial markets. Finally, the ADF test results show that the return series of all countries are stationary at the 1% significance level, meeting the requirements for further econometric analysis.

**Table 2:** Descriptive Statistics of stock index returns.

Country	Mean	Median	Max	Min	Std_Dev	Skewness	Kurtosis	JB_Stat	ADF_Stat
Argentina	0.0015	0.0008	0.2057	-0.4769	0.0228	-1.9860	40.6171	371015.7081	-17.8878 ${\;}^{***}$
Australia	0.0001	0.0004	0.0677	-0.1020	0.0101	-0.7348	8.9940	18504.1269	-17.5667 ${\;}^{***}$
Brazil	0.0004	0.0000	0.1368	-0.1599	0.0162	-0.4030	9.4842	20186.0283	-16.6449 ${\;}^{***}$
Canada	0.0002	0.0005	0.1129	-0.1318	0.0103	-1.0352	22.6914	115659.8139	-17.8163 ${\;}^{***}$
China	0.0002	0.0005	0.0903	-0.0926	0.0148	-0.4901	5.2177	6280.7607	-15.6971 ${\;}^{***}$
France	0.0001	0.0003	0.1059	-0.1310	0.0131	-0.2607	8.9112	17753.3523	-17.8328 ${\;}^{***}$
Germany	0.0003	0.0006	0.1080	-0.1305	0.0131	-0.2047	8.3772	15673.4869	-18.0061 ${\;}^{***}$
India	0.0005	0.0003	0.1599	-0.1410	0.0132	-0.3483	13.4480	40398.7288	-16.5273 ${\;}^{***}$
Indonesia	0.0005	0.0003	0.0970	-0.1095	0.0120	-0.5807	9.0101	18388.0821	-16.3324 ${\;}^{***}$
Italy	0.0000	0.0005	0.1087	-0.1854	0.0145	-0.7318	11.5208	30048.1068	-17.1955 ${\;}^{***}$
Japan	0.0003	0.0000	0.1323	-0.1323	0.0140	-0.5442	9.5046	20391.3688	-17.5741 ${\;}^{***}$
South Korea	0.0002	0.0003	0.1154	-0.1090	0.0129	-0.3768	7.0226	11115.4053	-17.7203 ${\;}^{***}$
Mexico	0.0004	0.0002	0.1044	-0.0727	0.0113	-0.0137	6.6486	9849.8953	-17.6986 ${\;}^{***}$
Russia	0.0003	0.0004	0.2523	-0.4047	0.0191	-1.9472	57.4087	737536.9322	-17.6681 ${\;}^{***}$
Saudi Arabia	0.0003	0.0000	0.0905	-0.1033	0.0108	-0.9773	17.5773	69680.7814	-17.5875 ${\;}^{***}$
South Africa	0.0003	0.0001	0.0791	-0.1045	0.0125	-0.2224	4.9800	5570.8029	-18.0235 ${\;}^{***}$
Turkey	0.0007	0.0008	0.1213	-0.1334	0.0169	-0.5134	5.1580	6163.7277	-16.7529 ${\;}^{***}$
UK	0.0000	0.0002	0.0938	-0.1151	0.0108	-0.3951	11.2408	28290.2889	-18.6789 ${\;}^{***}$
USA	0.0003	0.0004	0.1096	-0.1277	0.0116	-0.4941	13.5724	41257.2415	-17.9193 ${\;}^{***}$

## 5 Empirical analysis

### 5.1 Transfer entropy matrix

Based on the methodology introduced in Sect 3.2, we calculate the transfer entropy among the stock markets of the 19 G20 member countries over the full sample period to depict the transmission of risk information. The following results summarize the ranking of countries by risk outflow, inflow, net outflow, and net inflow.

Table 3 presents the results of the above analysis, ranking the risk inflows and outflows for G20 countries during the full sample period. Firstly, the United States exhibits the strongest risk output capability, ranking first in both risk information outflow and net outflow, demonstrating its systemic influence as a global financial center. Australia is the largest recipient of risk information, ranking first in both risk information inflow and net inflow, reflecting the high sensitivity of its market to international risks. Secondly, the United States, Canada, and Mexico dominate the top three positions for risk outflows, forming a significant regional risk output cluster.

**Table 3:** Risk information flow among countries.

Rank	Risk Outflow	Risk Inflow	Net Risk Outflow	Net Risk Inflow
1	USA (0.336)	Australia (0.5219)	USA (0.1972)	Australia (0.3833)
2	Canada (0.3226)	Japan (0.3814)	Canada (0.1944)	Japan (0.2747)
3	Mexico (0.2479)	South Korea (0.3276)	Germany (0.1235)	South Korea (0.2066)
4	Germany (0.2155)	Indonesia (0.2176)	Mexico (0.1167)	Indonesia (0.1112)
5	UK (0.2046)	South Africa (0.192)	Brazil (0.0969)	South Africa (0.0341)
6	Brazil (0.2023)	India (0.1765)	France (0.091)	India (0.0309)
7	France (0.1965)	USA (0.1388)	Italy (0.0861)	China (0.0142)
8	Italy (0.1694)	Mexico (0.1311)	UK (0.0813)	Saudi Arabia (0.0049)
9	South Africa (0.1579)	Canada (0.1282)	Argentina (0.0534)	Turkey (-0.0024)
10	India (0.1456)	UK (0.1234)	Russia (0.0169)	Russia (-0.0169)
11	Australia (0.1386)	France (0.1055)	Turkey (0.0024)	Argentina (-0.0534)
12	South Korea (0.1209)	Brazil (0.1054)	Saudi Arabia (-0.0049)	UK (-0.0813)
13	Russia (0.1188)	Russia (0.1018)	China (-0.0142)	Italy (-0.0861)
14	Argentina (0.1069)	China (0.0925)	India (-0.0309)	France (-0.091)
15	Japan (0.1066)	Germany (0.0921)	South Africa (-0.0341)	Brazil (-0.0969)
16	Indonesia (0.1064)	Turkey (0.0868)	Indonesia (-0.1112)	Mexico (-0.1167)
17	Turkey (0.0892)	Italy (0.0833)	South Korea (-0.2066)	Germany (-0.1235)
18	China (0.0783)	Saudi Arabia (0.0735)	Japan (-0.2747)	Canada (-0.1944)
19	Saudi Arabia (0.0686)	Argentina (0.0534)	Australia (-0.3833)	USA (-0.1972)

**Fig 1:**
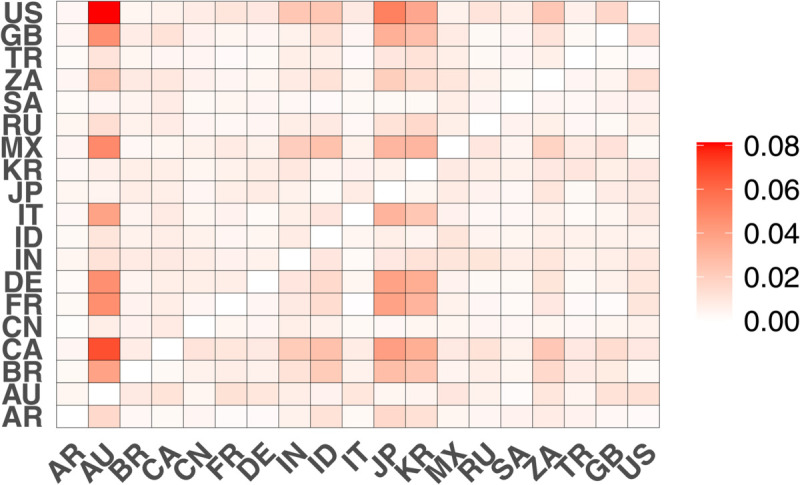
Heatmap of transfer entropy between G20 stock markets. The horizontal axis represents the target country (recipient of risk information), and the vertical axis represents the source country (provider of risk information).

For intuitive comparison, we use a heatmap to represent the transfer entropy between countries, as shown in Fig 1, which reflects the intensity of information flow between the stock markets of different countries. The direction of transfer entropy in the figure is from the vertical axis to the horizontal axis, with the axes labeled using the two-letter country codes of G20 countries. To facilitate comparison, the colors of all the heatmaps are adjusted to the same range. The horizontal axis of the heatmap represents the target country (the recipient of risk information), the vertical axis represents the source country (the provider of risk information), and the color intensity represents the magnitude of the transfer entropy. Red indicates higher intensity of information flow (stronger risk spillover), while white indicates lower intensity of information flow (weaker risk spillover), according to the legend.

From Fig 1, it can be seen that the United States, as the world’s largest economy, generally has higher transfer entropy values with other countries, indicating its significant informational influence in the global financial market. At the same time, Asian countries (such as Japan and South Korea) and European countries (such as Germany, France, the UK) also show relatively high transfer entropy values, reflecting the close interconnection of financial markets within the regions. Additionally, the asymmetry of the transfer entropy matrix indicates that information flow has directionality, with the United States having a significantly stronger informational influence on China than vice versa. Overall, the heatmap reveals the complex information transfer network between the world’s major economies, providing an important basis for understanding the interconnectedness of financial markets.

### 5.2 Risk spillover network of all samples

Based on complex network theory, the threshold method is used to construct a risk spillover network, representing the spillover effects between stock markets from a spatial dimension. This paper uses a multiple of the transfer entropy mean *μ* as the selection criterion for the threshold *θ*. Based on maintaining the overall volatility characteristics of the risk spillover, the evolution of the topological characteristics of the risk spillover network is studied.

**Fig 2:**
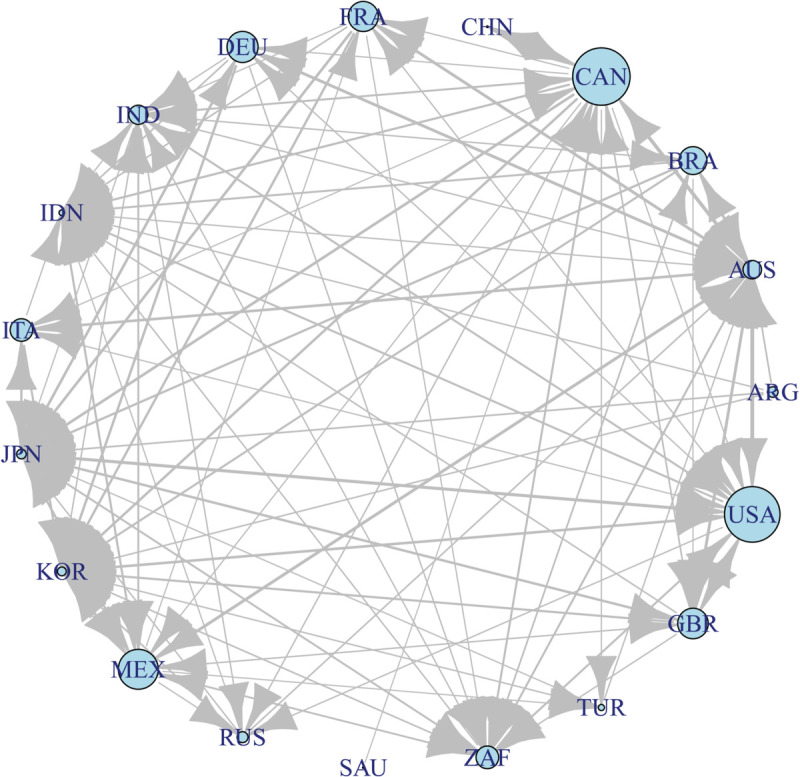
Directed weighted stock market network of the 19 G20 countries based on transfer entropy. Arrows indicate the direction of information flow between countries. Node labels represent the two-letter ISO country codes. Only edges exceeding the threshold for significant transfer entropy are shown.

In constructing the risk spillover network, the threshold *θ* is selected based on two principles: (1) ensuring all nodes remain connected without isolated nodes, and (2) maximizing the removal of weak links to reduce noise interference in the network structure. We tested the threshold parameter *k* in the range of 0.9–1.0. When θ=0.95μ the resulting network is fully connected, weak links with low transfer entropy are filtered out, the main risk spillover relationships are retained, key risk transmitter and receiver countries are clearly distinguishable, and the network visualization achieves an interpretable and informative structure suitable for subsequent analysis, as shown in Fig 2.

In the risk spillover network of the G20 stock markets, nodes are sized according to their weighted out-degree, reflecting each market’s risk output to others, while edges are weighted by transfer entropy, with thicker edges indicating stronger risk transmission. This representation clearly highlights the core position of the United States, which exhibits the highest risk spillover ability and transmits risk to multiple countries, including Canada, the UK, Germany, and France. Other important risk spillover countries, such as South Africa, India, and Australia, also play key roles in the Africa, Asia-Pacific, and global markets, respectively.

Germany, France, and the UK form the core of the European market, with strong risk transmission among them, and they also receive influences from North America and the Asia-Pacific region. Emerging market countries, such as India, Brazil, and South Africa, are both risk transmitters and receivers in the network, indicating that their market volatility increasingly impacts the global financial system. Mexico and South Africa are major risk receivers, absorbing market fluctuations from multiple countries, reflecting their markets’ high sensitivity to external shocks. China, Saudi Arabia, and Turkey occupy relatively peripheral positions in the network, with weaker risk spillover connections to the global market, demonstrating a certain degree of market independence.

PageRank is a network centrality algorithm originally developed by Brin and Page (1998) [54] to rank the importance of nodes in a network. In the context of the risk spillover network, PageRank reflects the importance of a market by considering not only the direct risk it receives from other markets, but also the indirect influence it can exert through the network structure. To implement PageRank, we construct a directed weighted network where each node represents a G20 country’s stock market, and the edges represent risk transmission. The edge weights are given by the Transfer Entropy (TE) values computed in Sect 3, which measure the directional information flow between markets.

In this weighted network, a market connected to highly influential markets will receive a higher PageRank score, capturing both direct and indirect risk propagation. We calculated the weighted PageRank for all 19 countries, ranked the countries based on this value, and for the top ten ranked countries, computed additional network metrics including weighted in-degree centrality (In_Strength), weighted out-degree centrality (Out_Strength), and weighted betweenness centrality, as shown in Table 4.

**Table 4:** Network topology indicators of the top 10 countries in PageRank ranking. Maximum and minimum values of each indicator are highlighted in bold.

Market	In_Strength	Out_Strength	Weighted_PageRank	Weighted_Betweenness
Australia	**0.4999**	0.0982	**0.1464**	**110**
Japan	0.3589	0.0510	0.0990	1
South Korea	0.3073	0.0467	0.0886	14
South Africa	0.1513	0.1206	0.0814	4
Mexico	0.0756	0.2124	0.0797	6
United States	0.1062	0.2957	0.0770	31
India	0.1225	0.1019	0.0618	2
Indonesia	0.1922	**0.0297**	0.0561	**0**
Canada	0.0738	**0.3055**	0.0544	49
United Kingdom	**0.0534**	0.1528	**0.0469**	10

The analysis results show that Australia, Japan, South Korea, and South Africa have relatively high PageRank values, indicating that they occupy core positions in the risk transmission network. In terms of market importance, Australia has the highest PageRank value, meaning it receives a significant amount of risk and may exert a strong impact on other markets in the G20. Japan and South Korea also rank relatively high, which is consistent with actual economic linkages in the global market. Regarding market influence, Japan and South Korea exhibit higher in-strength, while the United States shows a relatively low in-strength but a very high out-strength, suggesting that it functions more as a risk exporter rather than a passive recipient. In terms of intermediary markets, Canada and the United States record the highest betweenness values, showing that they play important bridging roles in the global financial risk transmission network, connecting multiple markets.

### 5.3 Risk spillover network of samples from different periods

Extreme events often lead to an increase in systemic risk, and the risk spillover patterns in financial markets can change significantly under different economic cycles or the influence of specific events. To identify the dynamic changes in risk spillover effects among stock markets of different countries during different events, we define the period from August 1, 2007, to March 31, 2009, as the global financial crisis period, from July 1, 2011, to December 31, 2013, as the European debt crisis period, from December 1, 2019, to December 31, 2022, as the COVID-19 pandemic period, and from February 1, 2022, to December 31, 2024, as the Russia-Ukraine conflict period, as shown in Table 5.

**Table 5:** Sub-periods of the full observed data set.

Sub-periods	Period 1	Period 2	Period 3	Period 4
Dates	2007.08-2009.03	2011.07-2013.12	2019.12-2022.12	2022.02-2024.12
Extreme events	Global financial crisis	European Debt Crisis	COVID-19	Russia-Ukraine conflict
Number of observations	406	606	729	694

**Fig 3:**
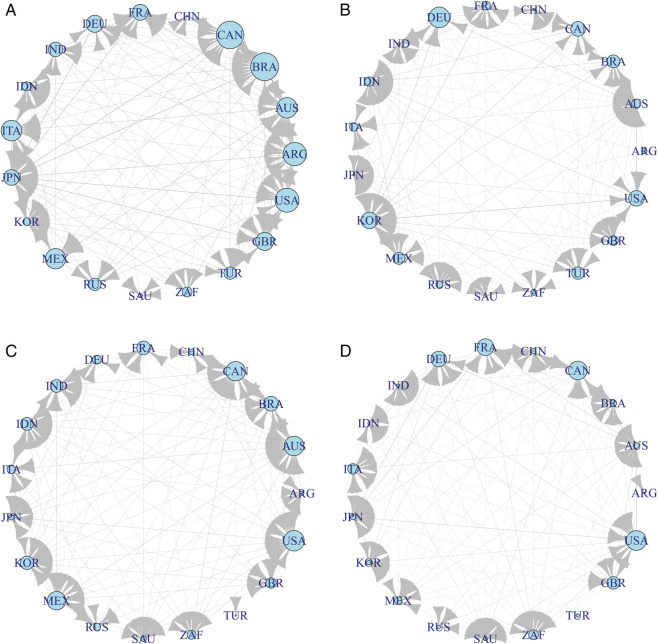
Risk spillover network under different extreme events.

In order to study the dynamic changes of risk spillover information during different periods, we calculated the transfer entropy of G20 countries’ stock markets during the four extreme event periods, and then constructed the risk spillover networks for each period. Fig 3a, 3b, 3c, and 3d represent the risk spillover networks during the global financial crisis period, the European debt crisis period, the COVID-19 pandemic period, and the Russia-Ukraine conflict period, respectively.

During the Global Financial Crisis (Period 1), countries such as Canada, Brazil, United States, Australia and Argentina had strong risk spillover relationships with other nations. There was a significant regional spillover effect among European countries. Similar regional spillover effects were observed among Asian countries (such as Japan, South Korea, and India), but they were not as widespread as in European countries.

During the European Debt Crisis (Period 2), Germany, United States, France and Russia exhibited the strongest risk spillover relationships. Germany had significant spillovers with Australia, Brazil, Canada, Italy, Japan, and South Korea, among others. Russia had many spillovers with countries like Australia, France, India, Italy, and Mexico. Among European countries, France, Italy, and Germany continued to show strong regional spillover effects.

During the COVID-19 Pandemic (Period 3), the effects of the pandemic were widespread, and the regional spillover relationships that existed during the previous two extreme events disappeared. Additionally, compared to the previous two extreme events, China’s participation in the global financial market increased, making it one of the few risk transmitters.

During the Russia-Ukraine Conflict (Period 4), most countries exhibited bidirectional spillover relationships. Countries such as France, Germany, Canada, Italy and the United States became major transmitters of risk, while countries like Brazil, Canada, and South Africa were major recipients of risk.

Finally, to examine the dynamic changes in the topology of the spillover risk network during extreme events, we calculated the in-degrees and out-degrees of the G20 countries’ stock market risk spillover networks during the four extreme event periods. Based on the previous static analysis of the risk spillover across the entire sample period, the analysis of each stock market’s risk reception or spillover intensity is shown in Table 6.

**Table 6:** In-degree and out-degree centrality measures across periods. Maximum and minimum values in each period are highlighted in bold.

Country	Period 1		Period 2		Period 3		Period 4	
	In-Degree	Out-Degree	In-Degree	Out-Degree	In-Degree	Out-Degree	In-Degree	Out-Degree
Argentina	11	12	**0**	5	5	4	1	**0**
Australia	**13**	12	**17**	9	**15**	13	10	7
Brazil	11	**14**	5	10	9	11	8	7
Canada	10	**14**	5	9	13	13	6	**14**
China	**3**	**1**	6	3	4	4	8	5
France	10	8	7	7	5	12	7	**14**
Germany	5	9	4	**16**	3	7	7	12
India	5	8	8	2	10	11	9	4
Indonesia	11	6	**15**	9	13	11	10	**2**
Italy	9	12	5	6	6	6	8	9
Japan	**14**	10	12	2	10	5	11	7
South Korea	12	5	14	**14**	12	11	9	5
Mexico	5	10	7	9	10	**13**	7	9
Russia	5	8	8	6	5	7	4	4
Saudi Arabia	**4**	**3**	5	**1**	11	5	**14**	4
South Africa	5	5	4	5	8	8	10	11
Turkey	7	6	6	12	**1**	**0**	**0**	5
United Kingdom	10	7	8	8	8	9	6	13
United States	11	11	5	8	**14**	12	9	12

According to the results in Table 6, the risk spillover network of the G20 stock markets exhibits a crisis-type-driven structure. During systemic crises (Periods 1 and 3), multiple markets such as Australia (13,12), Japan (14,10), and the United States (14,12) show high in-degree and out-degree, indicating multi-central spillovers. In contrast, during regional crises (Periods 2 and 4), the network becomes concentrated around a few dominant exporters, for example Germany (4,16) and South Korea (14,14) in Period 2, and Canada (6,14) and France (7,14) in Period 4. Emerging markets like India and Mexico also shift from mainly risk receivers (e.g., India 8,2 in Period 2) to secondary exporters (e.g., Mexico 10,13 in Period 3). This pattern confirms that systemic crises lead to multi-central spillovers, while regional crises are characterized by single-centered dominance.

## 6 Discussion

This study analyzes the risk propagation mechanisms of G20 stock markets from the perspectives of financial risk spillovers and complex network theory, revealing the nonlinear, directional, and dynamic characteristics of risk transmission. The empirical results indicate pronounced regional clustering in stock market risk spillovers, with stronger propagation occurring among markets within the same region. Developed markets primarily act as risk exporters. In contrast, emerging and Asian markets tend to serve as risk receivers or bridging nodes, reflecting their structural positions and heterogeneous capacities to absorb external shocks. The impacts of different extreme events (the global financial crisis, the European debt crisis, the COVID-19 pandemic, and the Russia-Ukraine conflict) vary across regions and periods, with developed economies experiencing stronger initial shocks and resource-based and emerging markets displaying higher vulnerability.

From a theoretical perspective, integrating transfer entropy with complex network analysis enables the capture of nonlinear and directional dependencies that traditional linear models cannot identify. It also facilitates the identification of key risk nodes and core risk-transmitting markets. These findings not only enhance our theoretical understanding of risk transmission but also offer practical implications for market participants and regulators. Practically, identifying key nodes provides guidance for investors to optimize asset allocation and adjust risk exposure. For regulators, network-based analysis helps to detect regions of concentrated systemic risk and potential contagion pathways, offering scientific support for cross-border capital flow management, early warning systems, and macroprudential policy implementation. During global extreme events, monitoring the dynamic evolution of the risk propagation network allows regulators to identify high-risk markets and bridging nodes, enabling targeted risk prevention and control measures.

## 7 Conclusions

This study employs the transfer entropy method to construct a spillover index matrix for G20 stock markets, identifying the roles and positions of each country or region within the risk spillover system from a static perspective. Additionally, risk spillover networks are constructed for the global financial crisis, the European debt crisis, the COVID-19 pandemic, and the Russia-Ukraine conflict, allowing for a comparison of risk propagation differences under various extreme events. The results indicate clear regional clustering in risk spillovers, with the United States and European markets primarily serving as net risk exporters, while Australia and Asian markets mainly act as net risk receivers. Emerging markets often function as bridging nodes. The impacts of extreme events differ across regions: the global financial crisis had the greatest effect on European and U.S. markets; the European debt crisis mainly affected Europe but also transmitted moderate risks to developed Asian countries such as Japan and South Korea; and the COVID-19 pandemic and Russia-Ukraine conflict exposed resource-based countries to higher risk.

The main contributions of this study include:

- **Methodological innovation**: Integrating transfer entropy with complex network theory to provide a nonlinear, directional, and structured analysis of stock market risk spillovers;
- **Empirical application**: Systematically comparing spatial and dynamic differences in risk spillovers across extreme events and identifying key risk nodes;
- **Policy implications**: Revealing the roles of different markets in risk transmission, offering guidance for portfolio management, risk monitoring, and cross-border financial regulation.

This study has several limitations, including constraints in data frequency and market coverage, as well as the exclusion of micro-level institutional behaviors that may affect risk transmission. Future research could incorporate high-frequency data, additional markets, and multi-layer network analysis to further enrich the understanding of global financial risk propagation mechanisms.
